# Effect of Iignocellulosic Nanoparticles Extracted from Yerba Mate (*Ilex paraguariensis*) on the Structural, Thermal, Optical and Barrier Properties of Mechanically Recycled Poly(lactic acid)

**DOI:** 10.3390/polym12081690

**Published:** 2020-07-29

**Authors:** Freddys R. Beltrán, Marina P. Arrieta, Gerald Gaspar, María U. de la Orden, Joaquín Martínez Urreaga

**Affiliations:** 1 Dpto. Ingeniería Química Industrial y Medio Ambiente, Universidad Politécnica de Madrid, E.T.S.I. Industriales, 28006 Madrid, Spain; geraldmanuel.gaspar@upm.es (G.G.); joaquin.martinez@upm.es (J.M.U.); 2 Grupo de Investigación: Polímeros, Caracterización y Aplicaciones (POLCA), 28006 Madrid, Spain; mariula@ucm.es; 3 Dpto. Química Orgánica, Facultad de Óptica y Optometría, Universidad Complutense de Madrid, 28037 Madrid, Spain

**Keywords:** poly(lactic acid), mechanical recycling, yerba mate, bionanocomposites

## Abstract

In this work, yerba mate nanoparticles (YMNs) were extracted from *Ilex paraguairiencis* yerba mate wastes and further used to improve the overall performance of mechanically recycled PLA (PLAR). Recycled PLA was obtained by melt reprocessing PLA subjected to an accelerated ageing process, which involved photochemical, thermal and hydrothermal ageing steps, as well as a final demanding washing step. YMNs (1 and 3 wt. %) were added to the PLAR during the melt reprocessing step and further processed into films. The main goal of the development of PLAR-YMNs bionanocomposites was to increase the barrier properties of recycled PLA, while showing good overall performance for food packaging applications. Thus, optical, structural, thermal, mechanical and barrier properties were evaluated. The incorporation of YMNs led to transparent greenish PLAR-based films with an effective blockage of harmful UV radiation. From the backbone FTIR stretching region (bands at 955 and 920 cm^−1^), it seems that YMNs favor the formation of crystalline domains acting as nucleating agents for PLAR. The morphological investigations revealed the good dispersion of YMNs in PLAR when they are used in the lowest amount of 1 wt. %, leading to bionanocomposites with improved mechanical performance. Although the addition of high hydrophilic YMNs increased the water vapor transmission, the addition of 1 wt. % of YMNs enhanced the oxygen barrier performance of the produced bionanocomposite films. These results show that the synergistic revalorization of post-consumer PLA and nanoparticles obtained from agri-food waste is a potential way for the production of promising packaging materials that meet with the principles of the circular economy.

## 1. Introduction

The development of bioplastics has raised a fair amount of interest in recent years. This is due to the constant growth of the consumption of fossil-fuel based plastics, which is leading to important environmental and raw materials availability problems. Among the most important bioplastics is poly(lactic acid) (PLA), which is an aliphatic polyester produced from renewable resources. PLA is obtained, on an industrial scale, via the ring-opening polymerization of lactide, the cyclic dimer of lactic acid, which is in turn produced by the fermentation of carbohydrates present in renewable feedstock such as corn, sugar beet or potato [1,2]. Due to its intrinsic biocompatibility and biodegradability, PLA was initially developed with a focus on biomedical applications. However, the development of new grades with improved thermal, mechanical and optical properties has turned PLA into one of the most important bioplastics on the market, with applications on several industrial sectors, such as the textile, automotive and especially in short-term applications, such as those coming from the food packaging sector [3,4]. This wide variety of applications is leading to a continuous growth on the production of PLA, reaching a global production capacity of 270 kt in 2019 [5].

The use of PLA in applications commonly dominated by fossil-fuel-based plastics, such as packaging, could lead to important advantages from the sustainability point of view. However, it is worth noting that a massive use of PLA might result in some environmental problems. The newer grades of PLA, designed with demanding applications in mind, are very resistant and are only biodegradable at specific industrial conditions (i.e., 58 °C, RH% ≅ 65, pH ≅ 7.5, *C*/*N* relationship between 20:1 and 40:1) [6,7], which are not available in the environment (i.e., landfill) [8,9]. Hence, an inadequate management of the generated residues could lead to the accumulation of PLA wastes. Furthermore, the transition to a circular economy model has to be considered. In this circular economy model, plastics play a prominent role, as it can be seen from the strategies and directives proposed by the European Commission, including the need to replace single-use plastics by the end of 2020 [10,11,12]. These policies promote not only the reduction of plastic waste, but also the recovery of such wastes in order to reuse them, retaining their value. Therefore, the incorporation of PLA into the circular economy model constitutes a major challenge, which could be achieved through the mechanical recycling of PLA-based plastic wastes [13].

Although mechanical recycling allows for reducing the consumption of raw materials and the emissions related to the manufacture of PLA, previous studies [14,15,16] point out that mechanical recycling promotes chain scission reactions in PLA, resulting in a decrease of the molecular weight and of some important properties such as thermal stability and Vickers hardness. Therefore, the development of cost-effective and environmentally friendly methods to recover the properties of mechanically recycled PLA, and thus improve its recyclability, is a key challenge. In this regard, several alternatives have been proposed as valid approaches to increase the overall performance of recycled PLA, such as the use of thermal treatments [17], reactive extrusion with cross-linking agents and chain extender additives [18,19] and the use of inorganic fillers [3]. Furthermore, to guarantee the packaging green nature, another potential alternative is the utilization of reinforcements derived from renewable resources. For instance, in a recent work [20], the addition of small amounts of silk fibroin nanoparticles led to the improvement of thermal, mechanical and gas barrier properties of recycled PLA. It is widely known that nanocomposites show excellent mechanical, thermal, and gas barrier properties compared with the conventional polymeric materials or composites, [21,22]. Thus, the use of natural reinforcements for the development of PLA-based nanocomposites, intended for food packaging applications, represents a good option to improve the overall performance of PLA and recycled PLA (PLAR), without influencing the transparency which is very important for consumers acceptance [3,22,23].

From a circular economy point of view, it would be interesting to evaluate upgrading methods that also allow to valorize other wastes, such as those coming from agri-food or textile industries. In this regard, lignocellulosic residues from agri-food products are mainly considered as waste or low-value by-product [24,25,26]. Nevertheless, other lignocellulosic biomass derivatives have been recognized as optimal reinforcing fillers for the bioplastic industry due to the fact that they are biobased, light, stiff as well as non-abrasive for the plastic processing machinery [27,28,29]. In this context, several lignocellulosic nanoparticles have shown their ability to enhance the PLA overall performance, in terms of thermal, mechanical and barrier properties while also providing some anti-oxidant properties, thus increasing its interest in food packaging applications [27,29,30,31]. Polymer nanocomposites refer to multiphase polymeric systems where at least one of the constituent phases, commonly the nanofiller, has at least one dimension in the nanoscale range (<100 nm) [22]. The nanoparticles dimensions and properties depend on the raw material utilized for the extraction and the chemical process selected for their production [28]. A simple and aqueous extraction procedure to obtain lignocellulosic nanoparticles from yerba mate waste was recently proposed [32]. Yerba mate (*Ilex paraguairiensis*, Saint Hilaire) tree originates from the subtropical region of South America, and naturally grows in a limited zone within Argentina, Brazil and Paraguay. It is generally consumed as infusion due to its good taste and well-known antioxidant properties [32,33]. Yerba mate is composed from about 35% α-cellulose [34], 25% hemicellulose [34] and 25–30% lignin [34,35]. The presence of lignin results in yerba mate containing different amounts of polyphenols (i.e., caffeic and chlorogenic acids) [33], xanthines (i.e., caffeine and theobromine), flavonoids (i.e., catechin, quercetin, kaempferol and rutin) [36,37], amino acids, saponin and tannins as well as some vitamins (i.e., C, B_1_, and B_2_) [36,38]. Nowadays, Brazil is the largest producer of Mate (around 350 kt annually) [39], followed by Argentina, which produced 270 kt in 2019 [40], and Paraguay (around 100 kt annually). Its high consumption leads to the generation of a high amount of yerba mate wastes, since, after being used as infusion, it is wasted without any kind of revalorization [32,41]. Thus, their use for lignocellulosic nanoreinforcements production could not only provide a sustainable revalorization to such waste, as it was already demonstrated for virgin PLA [32], but it could also potentially help to recover the properties of mechanically recycled PLA by developing bionanocomposites with interest in the food packaging field. In fact, as yerba mate is a rich source of polyphenols, which display an antiradical activity similar to pure gallic acid (20 mg/mL) [38], it has gained interest as sustainable additive that could be used to improve and modulate the properties of biopolymers. For instance, yerba mate extract provided a significant improvement of a starchy polymeric matrix stability in acidic and alkaline media [37]. Moreover, yerba mate extract has been added to starch treated by hydrostatic pressure to increase the loading capacity, obtaining interesting carriers for antioxidants, in which the antioxidant activity was maintained after the high pressure treatment without changing the yerba mate polyphenols profile [33]. Similarly, yerba mate has been encapsulated into electrospun zein fibers, improving the thermal stability and proving antioxidant activity, and thus showing interest as antioxidant releasers for food packaging applications [42]. Lignocellulosic yerba mate nanoparticles (YMNs) has also been added to PLA (in 5 wt. %), showing that the high amount of polyphenols protects the polymeric matrix from the thermal degradation during processing, and yielding bionanocomposites with significantly improved mechanical performance, although they showed a somewhat green tonality [32].

The main objective of the present research is to study the effects of lignocellulosic nanoparticles extracted from yerba mate wastes on the properties of mechanically recycled PLA, aiming to revalorize both yerba mate and PLA wastes by developing high-performance bionanocomposites intended for food packaging applications. Yerba mate nanoparticles (YMNs) were obtained by means of an aqueous extraction procedure, followed by two filtration steps, following a previously optimized recipe [32]. Recycled PLA (PLAR) was obtained by subjecting PLA to an accelerated ageing process previously optimized [14], which involved photochemical, thermal and hydrothermal ageing steps, as well as a final demanding washing step to simulate the washing conditions used on an industrial recycling level. The bionanocomposites were prepared by extrusion followed by a compression molding process. The YMNs were previously freeze dried to obtain a powder. Considering the high amount of –OH on the surface of lignocellulosic nanoparticles, which induces high attraction between them, particularly during the freeze-drying process, the nanoparticles were characterized by means of dynamic light scattering (DLS) and Transmission Electron Microscopy (TEM), before and after the freeze-drying process. The structure of the recycled PLA reinforced with yerba mate nanoparticles was characterized using infrared (FTIR) and UV-visible spectroscopic techniques, Differential Sacanning Calorimetry (DSC), Scanning Electron Microscopy (SEM) and intrinsic viscosity (IV) measurements. The effect of the nanoparticles on the thermal stability was measured using Thermogravimetric analysis (TGA), while the mechanical performance was evaluated by nanoindentation measurements. Finally, regarding the potential application in the packaging field, special attention was given to the gas barrier performance, which is of critical importance in food packaging applications. Thus, the permeability to oxygen gas and water vapor of the obtained materials was measured and compared. The results show that the yerba mate nanoparticles can significantly enhance the barrier to oxygen in the recycled material.

## 2. Materials and Methods 

### 2.1. Materials

PLA, under the commercial name Ingeo^TM^ 2003D, was purchased from NatureWorks (Minnetonka, MN, USA). This grade presents a melt mass-flow rate of 6 g/10 min (2.16 kg at 210 °C). The yerba mate (*Ilex paraguariensis*) residue was collected after yerba mate infusion (Taragüi, Argentina) consumption in our own laboratory.

### 2.2. Nanoparticle Extraction from Yerba Mate Residues

**Figure 1:**
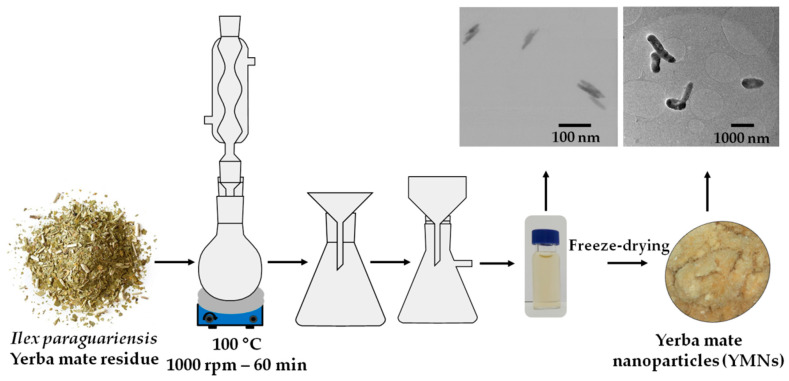
Schematic representation of mate nanoparticles’ extraction procedure.

The lignocellulosic-based nanoparticles, named yerba mate nanoparticles (YMNs), were obtained from yerba mate infusion wastes following an already developed recipe [32]. In brief, the residue of yerba mate infusion after its consumption was dried in an oven at 60 °C for 24 h. Then, 6 g of yerba mate infusion residue were mixed with 200 mL of distilled water and heated up to 100 °C under reflux during 60 min with vigorous magnetic stirring (1000 rpm). Next, the solid residue was eliminated by simple filtration, while the obtained mate extract solution was filtered off again (filter paper Whatman Grade 41:20–25 μm particle retention), frozen and further subjected to a freeze-drying process using a Flexi-Dry Freeze Dryer (FTS Systems, Stone Ridge, NY, USA) to obtain a powder as it is schematically represented in Figure 1. The obtained powder of YMNs was stored at 40 °C under vacuum during 24 h to remove any moisture before melt compounding process.

### 2.3. Preparation of the Samples

**Figure 2:**
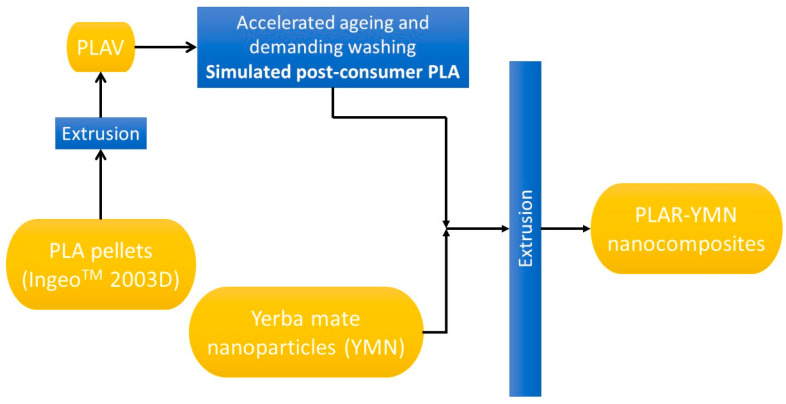
Procedure followed for the obtainment of the PLAR-YMN bionanocomposites.

The procedure followed for the ageing and subsequent obtainment of recycled PLA based materials is presented on Figure 2. Firstly, Ingeo 2003D pellets were processed by melt extrusion in a Rondol Microlab counter-rotating twin-screw extruder (Microlab, Rondol, France) with an *L*/*D* ratio of 20. The extrusion process was carried out at 60 rpm, using the following temperature profile (from hopper to die): 125, 160, 190, 190, 180 °C. The obtained material was transformed into films (thickness = 200 ± 10 µm) using an IQAP-LAP hot plate press (IQAP Masterbatch Group S.L., Barcelona, Spain) at 190 °C. Secondly, the films (PLAV) were subjected to an accelerated ageing process, consisting of the following stages: (i) 40 h of photochemical degradation using an ATLAS UVCON chamber (Chicago, IL, USA), equipped with eight F40UVB lamps; (ii) 468 h of thermal degradation in an oven at 50 °C and (iii) 240 h of hydrolytic degradation in distilled water at 25 °C. Thirdly, the aged samples were subjected to a demanding washing process, which was used in previous studies [14], using an aqueous solution of NaOH (1.0 wt. %) and Triton X (0.3 wt. %).

Lastly, the washed material was ground, and melt compounded together with yerba mate nanoparticles, in different proportions, at the same conditions used for the obtainment of PLAV films. Table 1 summarizes the different materials obtained in this study.

### 2.4. Characterization Techniques

The hydrodynamic size of YMNs were measured by means of a dynamic light scattering (DLS) analyzer. The obtained YMNs, in powder form, were dispersed in water (1 mg mL^−1^) by sonication and further measured at 20 °C in a Zetasizer Nano series ZS DLS equipment (Malvern Instrument Ltd., Malvern, UK).

YMNs were also observed by Transmission Electron Microscopy (TEM) in a JEOL JEM-1010 operating (JEOL Ltd., Tokyo, Japan) at 100kV. One droplet of YMNs aqueous suspension (1 mg mL^−1^) was deposited on carbon-coated copper grids and dried at room temperature during 20 min before TEM observation. The nanoparticles’ length and width were measured from the TEM images with ImageJ software; the mean and standard deviation of 15 nanoparticle measurements are reported.

Intrinsic viscosity measurements were performed, at 4 different concentrations in chloroform, at 25 °C in an Ubbelohde viscometer. All the solutions were filtered prior to the intrinsic viscosity measurement.

UV-Visible spectroscopy tests were conducted in a Varian Cary 1E UV-Vis spectrophotometer (Varian, Palo Alto, CA, USA) equipped with an integrating sphere and using a scanning speed of 400 nm/min. The overall transmittance in the visible region was then calculated according to the ISO 13468 standard.

Fourier transform infrared (FTIR) spectra of the different materials were recorded in Nicolet iS10 spectrometer (Thermo Fisher Scientific, Waltham, MA, USA), equipped with a diamond Attenuated Total Reflectance (ATR) accessory, using 16 scans and a resolution of 4 cm^−1^. The surface crystallinity degree (*X*_c_) of each nanocomposite was calculated from the absorbance of the band at 955 cm^−1^, measured in both the amorphous PLAR (*I*_0_) and the nanocomposite (*I*_f_), using Equation (1) [43]:

(1) $$X_{c}=\left(\frac{I_{0}-I_{f}}{I_{0}}\right)\times 100\%$$

The cryo-fractured surface microstructure of the cross section of each bionanocomposite film was observed by field emission scanning electron microscopy (FE-SEM) in a JEOL JSM 7600F microscope (JEOL Ltd., Tokyo, Japan). Films were previously sputtered with a gold layer to make them conductive.

Differential scanning calorimetry (DSC) scans were performed in a TA Instruments Q20 calorimeter (New Castle, DE, USA). Samples of 5 mg were placed in aluminum pans and subjected to the following protocol (under nitrogen atmosphere): (i) heating from 30 to 180 °C at 5 °C/min; (ii) isothermal step at 180 °C for 3 minutes; (iii) cooling from 180 to 0 °C at 5 °C/min; (iv) isothermal step at 0 °C for 1 min and (v) heating from 0 to 180 °C at 5 °C/min. 

Thermogravimetric analysis (TGA) was conducted on 10 mg samples using a TA Instruments TGA2050 thermobalance (New Castle, DE, USA). The samples were heated from 40 to 800 °C at 10 °C/min under nitrogen atmosphere. The onset degradation temperature (*T*_10_) was calculated at 10% of mass loss, and the maximum degradation temperature (*T*_max_) was determined from the peak of the first derivative of the TGA curve (DTG).

The water vapor transmission rate (WVTR) of the materials was measured, three times, by gravimetry according to the ISO 2525 standard. Thin films (9 ± 2 µm) of the samples were prepared by solvent casting from 0.01 g/mL chloroform solutions. The permeability cups were filled with 2 g of dry silica gel, sealed with the sample film and then placed in a desiccator with a saturated KNO_3_ solution at 23 °C (approximately 90% RH). The cups were weighed each hour for 6 h. WVTR (g/day cm^2^) was determined using Equation (2):

(2) $$WVTR=\frac{240*\left(m_{t}-m_{0}\right)}{A*t}$$

where *m_t_* is the mass of the cup at time *t*, *m*_0_ is the initial mass of the cup and *A* is the exposed area of the film.

Oxygen permeability tests were conducted at 30 °C in a homemade permeation cell, using a gas pressure of 1 kPa.

Nanoindentation tests were carried out using a Shimadzu DUH-211S dynamic Ultra-Microhardness Tester (Shimadzu Corporation, Kyoto, Japan), equipped with a Berkovich indenter. The measurements were conducted at room temperature (24.5 ± 0.5 °C), using a maximum load of 10 mN and a loading rate of 1.4632 mN/s. Maximum load was held for 5 s, and then it was retired. Each measurement was replicated 6 times.

## 3. Results and Discussion

### 3.1. Yerba Mate Nanoparticles’ Characterization

**Figure 3:**
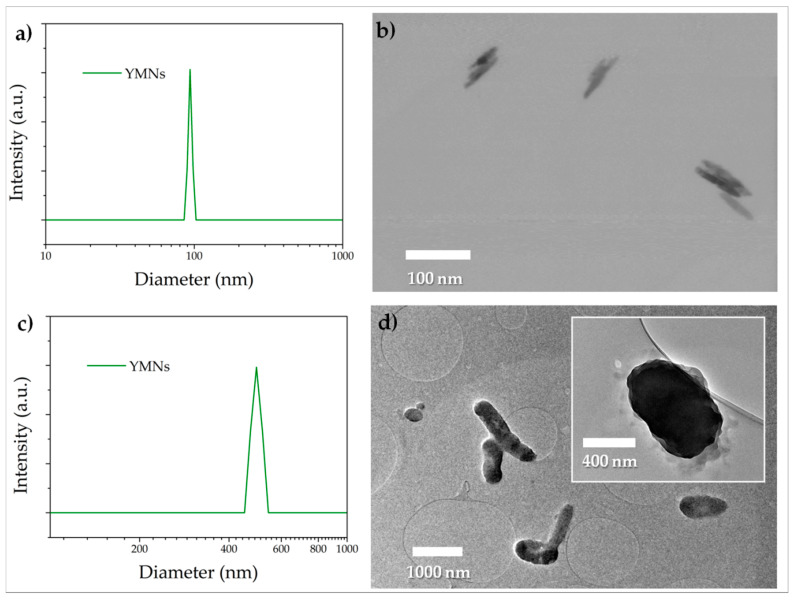
YMNs solution: (**a**) DLS measurements and (**b**) TEM images. YMNs powder: (**c**) DLS measurements and (**d**) TEM images.

The obtained mate extract solution after filtration was analyzed by DLS (Figure 3a), showing a monomodal size distribution from 85 to 103 nm, with an average size of 94 nm. There is a shoulder at higher sizes, around 500 nm, which has been related with the formation of agglomerates [32]. Considering that the DLS technique is designed to calculate the hydrodynamic diameter of spherical particles, the nanosize as well as the morphological aspect of the YMNs were further examined by TEM (Figure 3b). Individualized lignocellulosic 2D YMNs were observed. The yerba mate solution was then freeze-dried to obtain a powder, obtaining a yield of 19% ± 5%, which is in good agreement with previously reported work [32]. The size analysis of the obtained powder of yerba mate nanoparticles was also carried out by DLS (Figure 3c) and it revealed a monomodal size distribution with a dimension ranging from 450 to 545 nm, with an average size of 495 nm. From the TEM image of YMNs powder (Figure 3d), it can be seen that the YMNs tend to agglomerate due to the natural tendency of both, lignin and cellulose, to re-agglomerate and form strong hydrogen bonds as the water sublimate during freeze-drying process [44,45]. Nevertheless, they still showed sub-micron size with dimensions of 525 ± 136 in length and 302 ± 96 nm in width (see zoom in Figure 3d).

### 3.2. Structure and Morphology of the PLA-YMN Bionanocomposites

**Figure 4:**
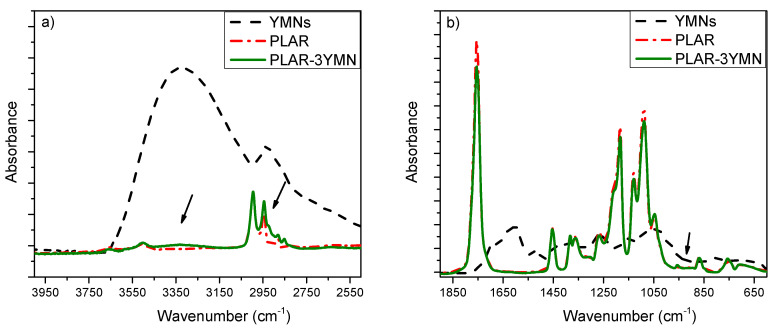
FTIR spectra of YMNs, recycled PLA (PLAR) and PLAR-3YMN bionanocomposite: (**a**) in the 4000–2500 cm^−1^ region and (**b**) in the 1900–600 cm^−1^ region.

The FTIR spectra of YMNs, PLAR and PLAR-3YMN are reported in Figure 4. The broad absorption band in the range of 3000–3700 cm^−1^ present in YMNs can be ascribed to the stretching vibration of the −OH groups in lignin as well as in cellulose molecules. The successful hemicellulose removal from yerba mate residue was confirmed by the absence of the band around 1730 cm^−1^ in YMNs [32], which corresponds to the acetyl and ester groups in hemicelluloses [46]. The spectrum that corresponds to PLAR-3YMM bionanocomposite shows a broad band at 3320 cm^−1^ (stretching vibration of the –OH groups) and a shoulder at 2920 cm^−1^ (C–H stretching vibration) (Figure 4a) that confirm the successful incorporation of YMNs in the recycled PLA [32]. The stretching vibration of the carbonyl group (–C=O) of PLA appears at 1750 cm^−1^ (Figure 4b) [47]. Moreover, the FTIR-ATR spectra of the bionanocomposites show very slight changes in the intensity of the bands at 920 and 956 cm^−1^ (Figure 4b). These absorptions have been assigned to skeletal C–C stretching mode coupled with CH_3_ rocking one [48,49,50]; while the band centered at 920 cm^−1^ corresponds to the 10_3_ helix chain conformation, characteristic of the crystalline forms, the band at 956 cm^−1^ is assigned to the amorphous phase. In this work, the crystallinity degrees in the surface of the nanocomposites were calculated from the absorbances of the band at 955 cm^−1^ in the different materials, using Equation (1). The values obtained, 16.7% for PLAR-3YMN and 12.5% for PLAR-1YMN indicate that the YMNs act as nucleating agents for recycled PLA.

**Figure 5:**
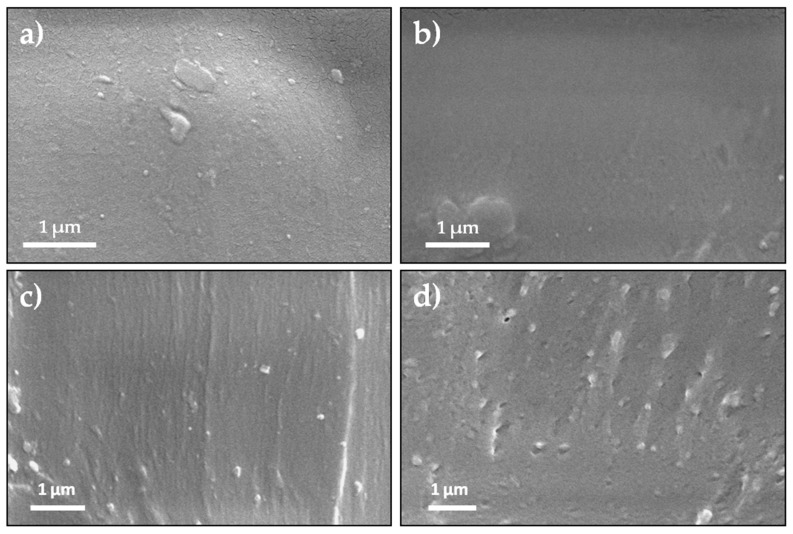
SEM observations of: (**a**) PLAV, (**b**) PLAR, (**c**) PLAR-1YMN and (**d**) PLAR-3YMN. (10,000× magnifications).

The effect of the addition of YMNs on the microstructure of mechanically recycled PLA was studied by means of SEM analysis. Neat virgin PLA (Figure 5a) shows the typical regular and smooth surface of an amorphous polymer. PLAR (Figure 5b) shows a very similar behavior than that of neat PLA (Figure 5a) with a rather more ductile pattern. This more plastic behavior could be ascribed to the already commented chain scission reactions that take place during the accelerated ageing and mechanical recycling, because the shorter polymer chains formed in these degradation processes plasticize the polymeric matrix. Meanwhile, PLAR-YMN bionanocomposites (Figure 5c,d) exhibited a rougher surface due to the YMNs’ reinforcing effect, as it has been already observed in virgin PLA blended with lignocellulosic nanoparticles [23,32].

The fracture surface depends on the concentration of YMNs (Figure 5c,d). In fact, in PLAR-1YMN bionanocomposite (Figure 5c), YMNs appear uniformly dispersed, with no phase separation between the nanoparticle and the polymeric matrix. However, in PLAR-3YMN (Figure 5d) some micro-holes can be seen, thus suggesting that YMNs in bionanocomposite containing 3 wt. % of YMNs show poor interfacial adhesion with PLAR matrix. Micro-holes have been already observed in virgin PLA reinforced with lignin nanoparticles and have been related to the formation of YMNs’ aggregates during bionanocomposite processing [29].

### 3.3. Properties of the PLA-YMN Bionanocomposites

#### 3.3.1. Effect of the Addition of YMNs on the Intrinsic Viscosity 

**Figure 6:**
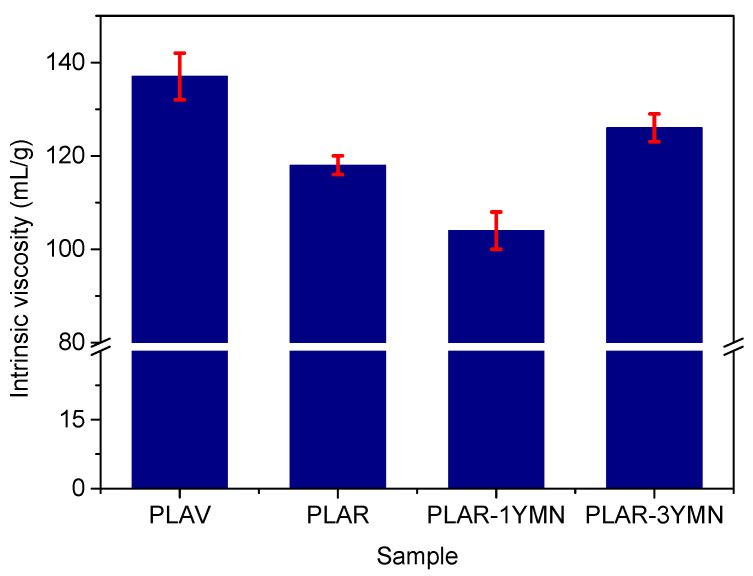
Intrinsic viscosity values of the different samples.

Intrinsic viscosity is related to the molecular weight of PLA, which plays a very important role in the final thermal and barrier properties of the materials. Furthermore, intrinsic viscosity is important from a processing point of view since industrial forming processes are frequently designed to operate at specific IV values. Thus, in order to get information regarding the effect of YMNs on the processing of PLAR-based bionanocomposites, the values of the intrinsic viscosity (IV) of PLAR in all the samples was determined by dissolving each sample in chloroform, followed by a filtration step to eliminate the YMNs. In accordance with previous works, Figure 6 shows that PLAR has an intrinsic viscosity around 14% lower than PLAV due to the degradation experimented [12,16] during the accelerated ageing, washing and reprocessing steps. 

Regarding the effect of the addition of yerba mate nanoparticles, Figure 6 shows that the material with only 1 wt. % of YMNs presents an intrinsic viscosity value 12% lower than PLAR. However, the sample with 3 wt. % of YMNs shows an intrinsic viscosity higher than that of the unfilled recycled material. This behavior might suggest that the addition of the nanoparticles produces two counteracting effects on the intrinsic viscosity of recycled PLA. On the one hand, the high hydrophilicity of the yerba mate nanoparticles might cause the absorption of small amounts of water during processing, which could result in a significant hydrolytic degradation of PLA during melt compounding. A similar behavior was observed in other PLA/lignocellulosic filler composites. For instance, Arrieta et al. [51] observed the reduction in the molecular weight of PLA bionanocomposites in virgin PLA reinforced with cellulose nanocrystals. Similarly, Way et al. [52] reported that PLA filled with lignocellulosic fibers showed a more severe degradation during melt processing than its unfilled counterpart. On the other hand, the antioxidant nature of yerba mate nanoparticles (due to the presence of phenolic compounds) could contribute to reduce the degradation of the polymer during extrusion, as it has been pointed out by Arrieta et al. [32] in a previous work. The results shown on Figure 6 indicate that at lower concentrations of YMNs, the negative effect of the hydrolysis prevails; however, at higher concentrations, the effect of the higher amounts of antioxidant compounds present in YMNs is more important, resulting in materials with higher IV values.

#### 3.3.2. Thermal Properties

**Figure 7:**
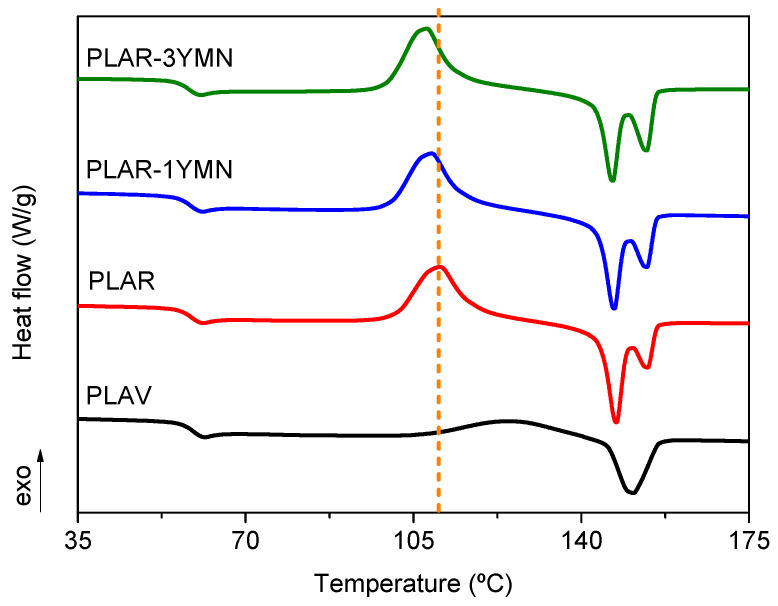
DSC scans corresponding to the second heating of the materials.

The effect of the addition of YMNs on the thermal properties of mechanically recycled PLA was studied by means of DSC and TGA. Figure 7 and Table 2 summarize the DSC results obtained for the different materials. As can be seen in Figure 7, PLAV show the characteristic thermal transitions of PLA: (i) a glass transition (*T*_g_) around 60 °C; (ii) a broad cold crystallization exothermic peak (*T*_cc_) above 100 °C and (iii) a melting endotherm (*T*_m_) centered at 150 °C. As for the behavior of PLAR, Figure 7 and Table 2 show that it has, overall, the same thermal transitions as PLAV. However, there are some noteworthy differences. Firstly, PLAR shows a narrower cold crystallization peak, which is also located at temperatures 15 °C lower than the virgin material. This difference could be attributed to the degradation of PLA during mechanical recycling, since the shorter polymer chains have increased mobility and crystallize more easily [14]. This behavior is also reflected in the higher values of the cold crystallization and melting enthalpies (∆*H*_cc_ and ∆*H*_m_ respectively) of PLAR. Secondly, Figure 7 shows that there are differences in the melting endotherm of the recycled material, since PLAR shows two well-defined melting peaks. This behavior has been reported in previous studies [53], and it has been attributed to the occurrence of a melt recrystallization phenomenon. Such a phenomenon consists of the melting of less ordered crystalline domains at lower temperatures, their rearrangement into crystalline structure as the temperature increases and a final melting of the more ordered crystals at a higher temperature. The fact that PLAR shows this behavior could also be explained by the degradation of the polymer during the recycling process. The shorter polymer chains present in PLAR could rearrange themselves during heating more easily due to their increased mobility and hence form more stable crystalline structure, which melt at higher temperatures.

Regarding the effect of the presence of the yerba mate nanoparticles, both Figure 7 and Table 2 show that the thermal behavior of PLAR-1YMN and PLAR-3YMN are very similar to that of mechanically recycled PLA. However, some differences can be seen in the cold crystallization temperature. Both Figure 7 and Table 2 show that the addition of the nanoparticles leads to a slight decrease in *T*_cc_ values. This behavior suggests that yerba mate nanoparticles act as nucleating agents, promoting the cold crystallization of PLA at lower temperatures, as it was seen by means of FTIR-ATR. The nucleating effect of different organic-based fillers has been previously reported by different authors, such as Fortunati et al. [44], Arrieta et al. [23] and Lizundia et al. [54] in PLA/cellulose nanocrystals bionanocomposites; as well as by Beltrán et al. [3] who studied recycled PLA/silk fibroin nanoparticles nanocomposites. It is also worth noting that PLAR-3YMN presents a melting behavior closer to PLAR than to PLAV, despite its higher IV value. This could also be explained by the nucleating effect of YMNs, since it allows for the occurrence of the melt recrystallization mechanism, despite the limited mobility of the longer polymer chains present in PLAR-3YMN. Nevertheless, the observed differences are rather small, thus indicating that the effect to the yerba mate nanoparticles on the thermal transitions of recycled PLA is limited.

**Figure 8:**
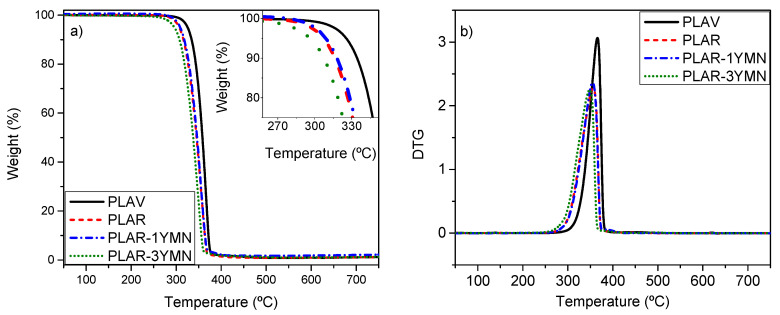
Dynamic (**a**) TGA and (**b**) DTGA curves of binary PLA nanocomposite films.

The effect of the recycling process as well as the addition of YMNs on the thermal properties of PLAR was also investigated by dynamic TGA measurements. The weight loss (TGA) and derivative (DTG) curves of virgin PLA (PLAV), recycled PLA (PLAR) and PLAR-YMNs bionanocomposites are reported in Figure 8, while the thermal parameters obtained from these curves are summarized in Table 2. All samples show a one-step degradation processes. While virgin PLA (PLAV) shows the highest maximum onset degradation temperature (*T*_10_ = 334.2 °C), PLAR-based samples presented a decrease in the thermal stability, as shown by the decrease in the onset degradation temperature, which has been ascribed to the presence of shorter polymer/oligomeric chains with lower thermal stability [14]. These results are in good agreement with the already commented reduction in the molecular weight when discussing the intrinsic viscosity measurements. In this context, Burgos et al. developed different PLA formulations plasticized with oligomeric lactic acid (OLA) and also observed a reduction of the onset degradation temperature, which decrease with increasing amounts of OLA [55]. The incorporation of 1 wt. % of YMNs did not promote significant changes in the thermal behavior of PLAR. While the *T*_10_ values slightly increased, the *T*_max_ remained almost invariable. However, with the incorporation of 3 wt. % of YMNs, both the *T*_10_ and the *T*_max_ decreased. These results may seem rather surprising considering that the material with 3 wt. % YMNs has a higher IV than PLAR. However, similar findings were observed by Fortunati et al. in virgin PLA reinforced with 1 and 3 wt. % of cellulose nanocrystals. They reported that the thermal stability of PLA decreased as the nanocellulose content increased and ascribed this behavior to the lower thermal stability of the cellulose nanocrystals (maximum degradation rate at about around 291°C) [30]. The DTG curve of the YMNs used in this work, which has been previously reported and analyzed [32], shows two maxima at 215 and 315 ° C, below the main PLA degradation temperature, so that the presence of 3 wt. % YMNs could explain the decrease in the thermal stability of the nanocomposite.

#### 3.3.3. Optical Properties

**Figure 9:**
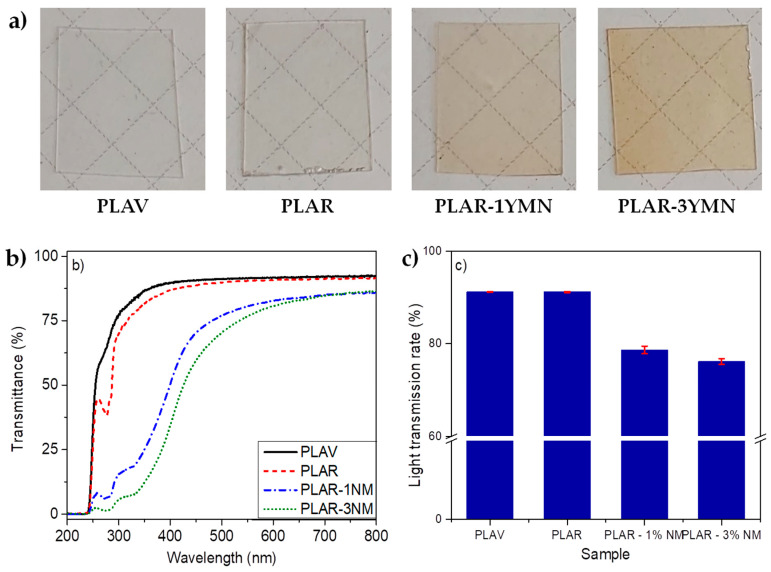
PLAV, PLAR and bionanocomposites (PLAR-1YMN and PLAR-3YMN): (**a**) visual appearance, (**b**) UV-vis spectra and (**c**) visible light transmission rates.

The visual appearances of virgin PLA, recycled PLA and YMN-reinforced bionanocomposites are shown in Figure 9a. From the visual appearance of the films, it is possible to observe that the recycled PLA remains transparent, with no apparent differences with virgin PLA. Meanwhile, bionanocomposites presented a somewhat green tonality, which was more evident in the case of the bionanocomposite with a higher amount of YMNs (PLAR-3YMN). In a previous work, virgin PLA has been reinforced with 5 wt. % of similar yerba-mate-based lignocellulosic nanoparticles and the developed films presented a brown tonality [32]. The transmission values in the visible (400–800 nm) and UV region of the spectra were determined by using UV-Vis spectroscopy (Figure 9b,c). 

The spectra show that films obtained from PLAV and PLAR are highly transparent in the visible region. In good agreement with the visual appearance of the films, the spectra show that the presence of YMNs leads to significant decreases in the visible light transmission (Figure 9b). The overall transmission rate in this spectral region falls from values higher than 90% in PLAV and PLAR to values clearly below 80% in biocomposites (78.6% and 76.1% of light transmission rates in PLAR-1YMN and PLAR-3YMN, respectively), although these materials remain transparent (Figure 9c). The presence of lignocellulosic aggregates in PLAR-3YMN decreased the visible light transmittance of the PLAR-based film, in good agreement with SEM analysis. Similar results have been observed in PLA/lignin nanoparticles bionanocomposites [31].

It is worth noting that PLAR shows lower UV light transmission than PLAV, with the appearance of a small absorption peak centered at 277 nm. This band is related with the formation of chain-end carboxyl groups, as a consequence of the degradation of the polymer that take place during the recycling process [20,56]. In the case of YMN-reinforced recycled plastics, this region is overlapped with different absorptions due to the polyphenols (i.e., chlorogenic acid, caffeic acid and rutin [33]) present in YMNs. 

The above spectra reveal that YMNs produce a strong UV blocking effect in the recycled PLA matrix. Other authors have already reported the UV blocking effect in virgin PLA reinforced with different lignocellulosic nanoparticles, such as in PLA/lignin nanoparticles bionanocomposites [31,57]; PLA/cellulose nanocrystals nanocomposites [51] and also in virgin PLA reinforced with 5 wt. % of similar yerba-mate-based lignocellulosic nanoparticles [32]. In the case of PLAR-1YMN film, the presence of only 1 wt. % of YMNs was able to block around 90% of UV-B and C, and this UV blocking effect was more marked in PLAR-3YMN, as could be expected.

In summary, it can be said that the addition of YMNs nanoparticles to the recycled PLA, in a proportion less than or equal to 3 wt. %, has an overall positive effect on the optical properties of the material. On the one hand, the transparency in the visible region is reduced, but the sheets of these bionanocomposites remain transparent, which is important in many cases of food packaging, because seeing the packed food through the packaging film is one of the most important requirements for consumers’ acceptance. On the other hand, the presence of YMNs greatly reduces UV transmission, thus slowing down the degradation of the contents of the container.

#### 3.3.4. Barrier Properties 

**Figure 10:**
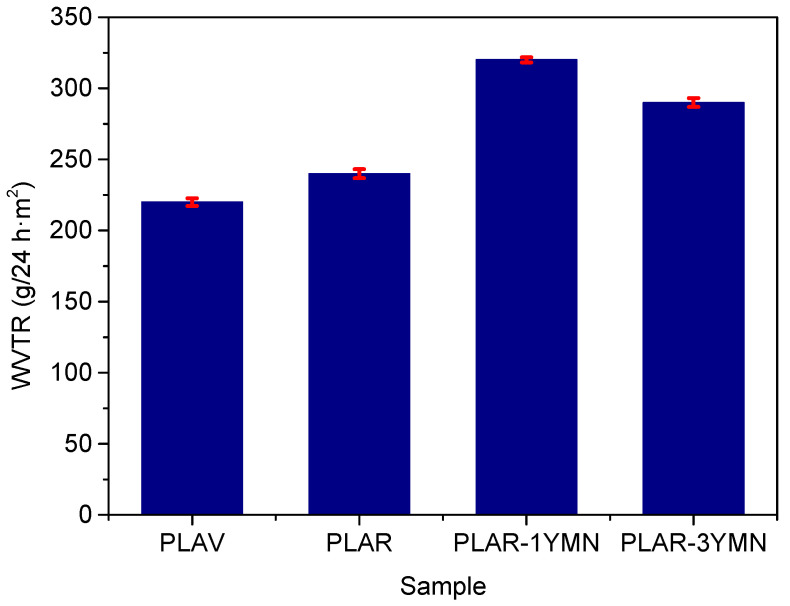
Water vapor transmission rate of the different materials.

**Figure 11:**
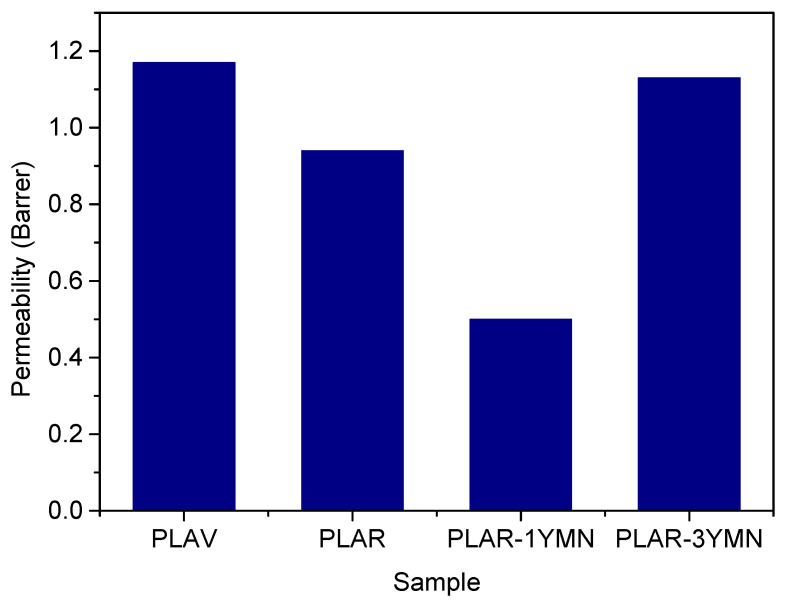
Oxygen permeability of the different materials.

The barrier properties against different gases are very important in food packaging applications, which is the most important market for PLA. Therefore, the effect of the addition of the YMNs on the gas barrier properties of mechanically recycled PLA was measured; the main results are reported in Figure 10 and Figure 11.

Figure 10 shows the WVTR of the different samples. The obtained values are similar to those reported in previous studies for PLA based materials [58]. It can be seen that mechanical recycling led to a slight increase in the WVTR of PLA. To explain this behavior, one should consider that the gas permeability, and hence the WVTR, of semicrystalline polymers depends on two factors: the diffusion coefficient and the solubility of the gas. These factors are affected by the molecular weight, structure and free volume of the polymer and by the temperature and nature of the gas molecules [12,29]. The observed increase in the WVTR of the mechanically recycled PLA could be related to the generation of terminal carboxyl and hydroxyl groups during the ageing and mechanical recycling, which decreases the hydrophobic character of the polymer, thus facilitating the passage of water vapor through the films. Regarding the effect of the nanoparticles, it can be observed that the nanocomposites show higher WVTR values than both unfilled PLAV and PLAR samples, which could be explained by the hydrophilic nature of the YMNs due to the high amount of –OH groups. In this context, Kim et al. [59] studied the WVTR of PLA reinforced with pristine lignin and acetylated lignin, reporting higher WVTR values for the PLA-lignin composites in comparison with neat PLA. This behavior was ascribed to the hydrophilic nature of pristine lignin. Meanwhile, acetylated lignin-based composites were able to decrease the WVTR values of neat PLA. Similarly, Espino-Perez et al. [60] developed PLA loaded with cellulose nanowhiskers (5, 14 and 30 wt. %), reporting that WVTR increased with the cellulose nanowhiskers content. This behavior was related to the hydrophilic nature of cellulose structures. In this work, PLAR-1YMN, the material with the lower amount of hydrophilic YMNs, shows higher WVTR than PLAR-3YMN, which can be related to the lower viscosity observed in PLAR-1YMN. This low viscosity, due to a stronger degradation, indicates the presence of more hydrophilic terminal groups in the polymer chains, which can explain the higher value of WVTR.

Figure 11 presents the oxygen permeability coefficient, measured in Barrer (1 Barrer = 3.35·10^−16^ mol m/m^2^ s Pa), of the different samples. It can be observed that the ageing and the mechanical recycling cause only a slight decrease in the oxygen permeability of PLA, despite the degradation observed by means of IV measurements. Similar results have been reported in a previous study [14] and have been attributed to the presence of two counteracting effects of the mechanical recycling on the permeability of PLA. On the one hand, the presence of shorter polymer chains might reduce the free volume inside the polymer, due to their better ability to rearrange themselves, reducing the diffusion coefficient. On the other hand, the generation of terminal –COOH and –OH groups during the ageing and recycling lead to an increase in the affinity between the polymer and the gas molecules, increasing the solubility of the gas into the polymer. The concurrence of these counteracting effects leads to the overall small changes observed in the oxygen permeability.

As for the behavior of the bionanocomposites, Figure 11 shows that the oxygen permeability is significantly reduced with the addition of 1 wt. % of YMNs. The reduction of the oxygen permeability due to the incorporation of cellulose nanocrystals have been already observed in virgin PLA/cellulose nanocrystals based bionanocomposites [51,61]. This behavior could be explained by the barrier effect caused by the dispersion of the nanoparticles in the polymer matrix, which leads to an increase in the tortuosity of the diffusion path traveled by the gas going through the polymer film. However, the oxygen permeability increased, reaching values close to those of unfilled PLA, when the amount of YMNs was 3 wt. %. It is well known that the tortuosity of the diffusion path depends on several factors (i.e., shape and aspect ratio of the filler, degree of dispersion or exfoliation, filler loading and orientation, adhesion to the matrix, moisture activity, filler-induced crystallinity, polymer chain immobilization, filler-induced solvent retention and porosity) [61]. Thus, this result could be due to the poor dispersion of the nanoparticles in the PLAR-3YMN sample, as was observed in SEM photographs. The poor dispersion of the nanoparticles might result in the formation of micro-pores in the polymer matrix, which act as low-resistance paths for the gas diffusion through the polymer. Therefore, this result underlines the success of the dispersion of low amounts of YMNs (1 wt. %) during melt-compounding process and its reinforcement effect produced in the final formulation.

#### 3.3.5. Mechanical Properties

**Figure 12:**
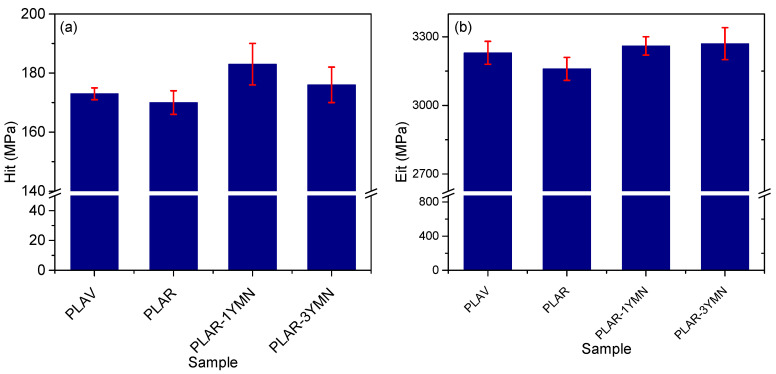
Indentation hardness (**a**) and Young modulus (**b**) of the different samples.

Mechanical properties play a very important role in food packaging applications; consequently, nanoindentation tests were conducted to determine the effect of the addition of YMNs on mechanically recycled PLA. Figure 12 shows the indentation hardness and the Young modulus of the different materials. It can be seen that both hardness and modulus values are in good agreement with those found in the literature for PLA samples [62,63]. It could also be seen that mechanical recycling led to a slight decrease in the mechanical properties of PLA, due to the degradation of the polymer during the ageing, washing and reprocessing steps. Similar results have been reported in previous works [14,62], who found that mechanical recycling led to small decrease in the mechanical properties of PLA.

Regarding the effect of the addition of the YMNs, it can be seen that samples with 1 wt. % YMN and 3 wt. % YMN show slightly higher values for hardness and modulus than unfilled PLAR. This result suggests that the presence of the YMNs nanoparticles has a reinforcing effect on the recycled PLA matrix. Similar trends have been reported in other PLA nanocomposites, for instance, Zaidi et al. [63,64] reported increases in both indentation hardness and the Young modulus with the addition of low amounts of organically modified montmorillonite. It is worth noting that, despite the overall improvement of the mechanical properties of recycled PLA with the addition of YMNs, better results are observed in the material with only 1 wt. % YMN. This behavior agrees with that observed in the oxygen permeability measurements and highlights the relevance of the better dispersion of lower amounts of YMNs.

## 4. Conclusions

The effect of the addition of lignocellulosic nanoparticles extracted from food waste, specifically yerba mate waste, on the structure, mechanical and barrier properties of mechanically recycled PLA (PLAR) was studied. PLAR was obtained by subjecting a commercial grade of PLA to accelerated ageing followed by mechanical recycling. Lignocellulosic yerba mate nanoparticles (YMNs) were extracted from yerba mate waste in an aqueous extraction process and added to PLAR in the reprocessing step at two levels (1 and 3 wt. %). FTIR and SEM analysis confirmed the successful incorporation of YMNs into the PLAR matrix. 

Ageing and mechanical recycling cause the degradation of PLA, leading to a decrease in the molecular weight, thermal stability and barrier performance. The addition of small amounts of YMNs significantly modifies some properties of the material, depending on the YMNs content. The nanoparticles act as nucleating agents, thus facilitating the crystallization of PLAR, without significantly reducing the average molecular weight. Although the nanoparticles slightly reduce the thermal stability of the material, due to their lower thermal stability, the material remains stable under processing conditions. Bionanocomposites with 1 wt. % of YMNs show a good dispersion of the nanoparticles; however, when the YMNs’ content rises up to 3 wt. %, although no phase separation was detected, YMNs tend to aggregate, inducing the formation of micro-voids. Thus, the addition of only 1 wt. % YMNs improved the mechanical performance and reduces oxygen permeability, a key property in food packaging materials. However, if the YMNs content rises to 3%, the effect on the oxygen barrier is negative, due to dispersion problems and the formation of micro-voids. In general, the incorporation of YMNs increases the water vapor transmission rate, due to the hydrophilic character of the nanoparticles. As for light transmission, another key property in food packaging, the addition of YMNs slightly reduces transmission in the visible region, but the recycled material remains transparent. However, nanoparticles dramatically reduce transmission in the UV areas of the spectrum, which can help slow down the degradation of the container’s content.

Overall, the results obtained indicate that the addition of yerba mate nanoparticles could lead to obtaining recycled PLA with good properties for the intended use and with significant improvements in some key properties, such as the barrier to UV light and oxygen. Considering that these nanoparticles are also obtained from a food residue and using an environmentally friendly extraction process, the use of YMNs could be the basis of a useful and potentially competitive method to improve the recyclability of PLA and other similar polymers.

## Figures and Tables

**Table 1:** Materials obtained after the recycling process.

Sample	Description
PLAV	PLA obtained after the first extrusion and compression molding steps
PLAR	PLA obtained after the accelerated ageing, washing, and melt compounding of PLAV
PLAR-1YMN	PLAR with 1 wt. % of yerba mate nanoparticles
PLAR-3YMN	PLAR with 3 wt. % of yerba mate nanoparticles

**Table 2:** DSC (second heating) results as well as TGA parameters of the different materials.

Sample	*T*_g_ (°C)	*T*_cc_ (°C)	*T*_m_ (°C)	∆*H*_cc_ (J/g)	∆*H*_m_ (J/g)	*T*_10_ (°C)	*T*_max_ (°C)
PLAV	59.0	125.2	150.8	14.5	15.4	334.2	365.9
PLAR	58.6	110.5	147.3–153.9	27.1	27.7	316.1	355.9
PLAR-1YMN	58.6	108.9	146.9–153.7	26.7	27.8	318.1	354.6
PLAR-3YMN	58.1	107.7	146.5–153.7	28.2	28.3	307.3	349.1
